# Impact of Antibiotics on the Proliferation and Differentiation of Human Adipose-Derived Mesenchymal Stem Cells

**DOI:** 10.3390/ijms18122522

**Published:** 2017-11-24

**Authors:** Aleksandra Skubis, Joanna Gola, Bartosz Sikora, Jolanta Hybiak, Monika Paul-Samojedny, Urszula Mazurek, Marek J. Łos

**Affiliations:** 1 Department of Molecular Biology, School of Pharmacy with the Division of Laboratory Medicine in Sosnowiec, Medical University of Silesia in Katowice, 40-055 Katowice, Poland; jgola@sum.edu.pl (J.G.); bartoszsikora90@gmail.com (B.S.); umazurek10@gmail.com (U.M.); 2 Department of Pathology, Pomeranian Medical University, 70-204 Szczecin, Poland; jhybiak@gmail.com; 3 Department of Medical Genetics, School of Pharmacy with the Division of Laboratory Medicine in Sosnowiec, Medical University of Silesia in Katowice, 40-055 Katowice, Poland; mpaul@sum.edu.pl; 4 Małopolska Centre of Biotechnology, Jagiellonian University, Gronostajowa 7A str., 30-387 Krakow, Poland; 5 LinkoCare Life Sciences AB, 583 30 Linköping, Sweden; 6 Centre de Biophysique Moléculaire, UPR4301 CNRS CS80054, Rue Charles Sadron, 45071 Orleans CEDEX 2, France

**Keywords:** adipose-derived stem cells, amphotericin B, copper (II) ions, osteogenesis, penicillin, streptomycin

## Abstract

Adipose tissue is a promising source of mesenchymal stem cells. Their potential to differentiate and regenerate other types of tissues may be affected by several factors. This may be due to in vitro cell-culture conditions, especially the supplementation with antibiotics. The aim of our study was to evaluate the effects of a penicillin-streptomycin mixture (PS), amphotericin B (AmB), a complex of AmB with copper (II) ions (AmB-Cu^2+^) and various combinations of these antibiotics on the proliferation and differentiation of adipose-derived stem cells in vitro. Normal human adipose-derived stem cells (ADSC, Lonza) were routinely maintained in a Dulbecco’s Modified Eagle Medium (DMEM) that was either supplemented with selected antibiotics or without antibiotics. The ADSC that were used for the experiment were at the second passage. The effect of antibiotics on proliferation was analyzed using the 3-[4,5-dimethylthiazol-2-yl]-2,5-diphenyltetrazolium bromide (MTT) and sulforhodamine-B (SRB) tests. Differentiation was evaluated based on Alizarin Red staining, Oil Red O staining and determination of the expression of ADSC, osteoblast and adipocyte markers by real-time RT-qPCR. The obtained results indicate that the influence of antibiotics on adipose-derived stem cells depends on the duration of exposure and on the combination of applied compounds. We show that antibiotics alter the proliferation of cells and also promote natural osteogenesis, and adipogenesis, and that this effect is also noticeable in stimulated osteogenesis.

## 1. Introduction

Adipose tissue is an excellent source of adult stem cells, which are called adipose-derived stem cells (ADSC) [1]. They hold great promise in cell replacement therapies, and studies demonstrated their properties in the regeneration of bones, joints or cardiomyocytes in vivo and in vitro [2,3,4]. The cell harvesting procedure is minimally invasive and it permits a large number of cells with strong proliferative ability and multidifferentiation potential to be extracted and then injected into bone, cartilage and adipose tissues. The ADSC are usually extracted from the SVF (stromal vascular fraction) that is obtained from adipose tissue and they have a fibroblast-like morphology and adhere to culture dishes [3,5,6,7].

Antibiotics are extremely important in transplantation and regenerative medicine procedures. Isolation and cultivation of adipose-derived stem cells usually involves the presence of the penicillin-streptomycin mixture [2,5,8,9] although some studies have also suggested using amphotericin B because of its widespread antifungal activity [10,11]. Gentamycin is also commonly used in cell cultures, although a penicillin-streptomycin (PS) mixture is the only one that is recommended by the American Type Culture Collection (ATCC) [12]. Protective/preventive antibiotic therapy improves the success/safety of transplantation procedures [10]. To minimize complications, stem cells must be isolated and cultured under the optimal conditions for a particular cell type, in order to use them in transplantology. They have to be maintained in an undifferentiated state to preserve their self-renewal potential. Unfortunately, it has been suggested that the antibiotics in a cell culture may change the regenerative potential and other biologic properties in many types of cells [13].

One of the most effective antifungal compounds is amphotericin B (AmB), a polyene antimycotic drug that belongs to the macrolides [14,15]. Due to the cytotoxic effect on human cells, its usefulness in stem cells cultivation may be problematic, however, there are available less toxic forms of the AmB. One of them is complex of amphotericin B with copper (II) ions (AmB-Cu^2+^), which was reported to be less toxic to animal cells and more potent in combating fungal infections [16,17,18,19]. Thus, replacing AmB with its modified form AmB-Cu^2+^ in stem cell culture may favorably affect their ability to proliferate and differentiate.

The influence of penicillin, streptomycin, amphotericin B and AmB-Cu^2+^ on stem cells, especially related to their impact on ADSC, has not been studied so far. The object of the present study was to evaluate the influence of these antibiotics on the growth and differentiation potential of ADSC. In the first step the influence of antibiotics and their combinations on stem cells viability was tested. Next, the potential of adipogenesis and osteogenesis promotion by antibiotics in the absence of dedicated differentiation factors was assessed (in basic medium). Obtained results have prompted us to the evaluation of the influence of antibiotics and their combinations on differentiation into osteoblasts in the dedicated osteogenesis medium.

## 2. Results

### 2.1. Effect of Antibiotics on Cell Viability in the ADSC

**Figure 1:**
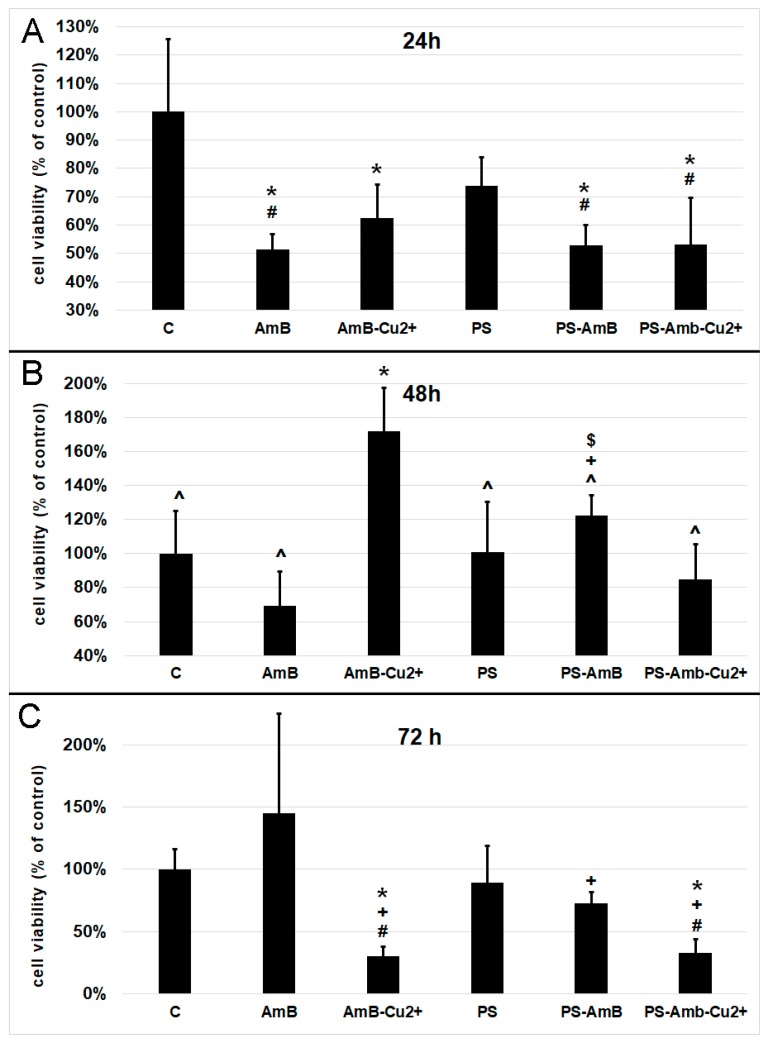
Cell viability based on the measurement of total cellular protein content after exposure to the tested antibiotics and their combinations for 24 (**A**), 48 (**B**) and 72 h (**C**). The bars represent the means ± standard deviation (SD) of the percentages of the control cell viability (100%); analysis of variance (ANOVA) with the Tukey post hoc test, * *p* < 0.05 vs. control, ^#^ *p* < 0.05 vs. PS, ^ *p* < 0.05 vs. AmB-Cu^2+^, ^+^ *p* < 0.05 vs. AmB, ^$^ *p* < 0.05 vs. PS-AmB-Cu^2+^.

The viability of cells was assessed indirectly by the measurement of total cellular protein content in ADSC after 24, 48 and 72 h of incubation with antibiotics. Cells treated for 24 h with AmB, AmB-Cu^2+^, PS-AmB and PS-AmB-Cu^2+^ showed a statistically significant decrease of viability comparing to the control (*p* = 0.0001, *p* = 0.0021, *p* = 0.0002, and *p* = 0.0001, respectively) (Figure 1A). Moreover, statistically significant decreased viability was also observed for cells treated with AmB, PS-AmB and PS-AmB-Cu^2+^ comparing to the PS-treated cells (*p* = 0.0226, *p* = 0.0431, *p* = 0.0217, respectively). 

After 48 h, cells treated with AmB-Cu^2+^ showed higher viability than the control (*p* = 0.0004), AmB (*p* = 0.0001), PS- (*p* = 0.0004), PS-AmB- (*p* = 0.0371) and PS-AmB-Cu^2+^-treated cells (*p* = 0.0001) (Figure 1B). PS-AmB caused higher cell viability comparing to AmB (*p* = 0.0082) and PS-AmB-Cu^2+^ (*p* = 0.0280).

Seventy-two hours of incubation caused a significant decrease in the viability of cells exposed to AmB-Cu^2+^ and PS-AmB-Cu^2+^, comparing to the control (*p* = 0.0145, *p* = 0.0206), PS (*p* = 0.0313, *p* = 0.0144), and AmB groups (*p* = 0.0001, *p* = 0.0002) (Figure 1C). AmB treatment increased the cell viability in comparison to PS-AmB group (*p* = 0.0104)

### 2.2. Effect of Antibiotics on the Mitochondrial Oxidative Activity of the ADSC

**Figure 2:**
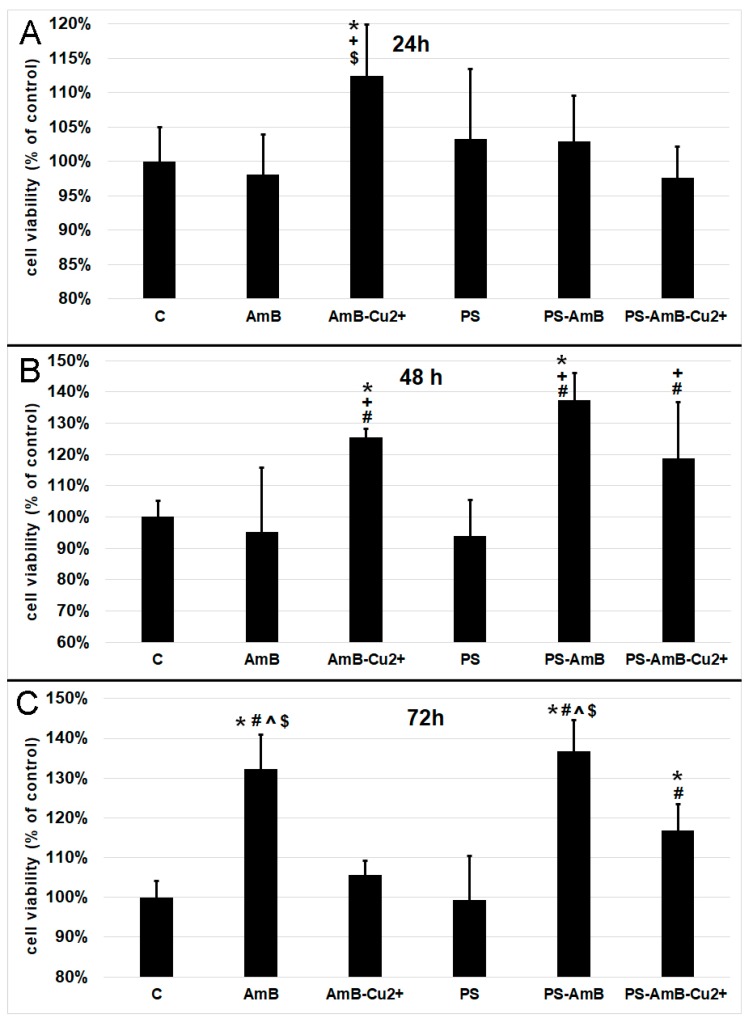
Cell viability based on the measurement of mitochondrial oxidative activity after exposure to the tested antibiotics and their combinations for 24 (**A**), 48 (**B**) and 72 h (**C**). The bars represent the means ± standard deviation (SD) of the percentages of the control cell viability (100%); ANOVA with the Tukey post hoc test, * *p* < 0.05 vs. the control, ^#^ *p* < 0.05 vs. PS, ^ *p* < 0.05 vs. AmB-Cu^2+^, ^+^ *p* < 0.05 vs. AmB, ^$^ *p* < 0.05 vs. PS-AmB-Cu^2+^.

Based on the results pertaining cell viability presented in Figure 1, we decided to measure oxidative activity of mitochondria by 3-[4,5-dimethylthiazol-2-yl]-2,5-diphenyltetrazolium bromide (MTT) assay. After 24 h, the AmB-Cu^2+^ treatment caused a statistically significant increase of mitochondrial oxidative activity comparing to the control (*p* = 0.0200), as well as cells exposed to AmB (*p* = 0.0049) and PS-AmB-Cu^2+^ (*p* = 0.0034) (Figure 2A). 

48 h exposure caused significant increase of mitochondrial activity of cells treated with AmB-Cu^2+^ and PS-AmB in comparison to control (*p* = 0.0334, *p* = 0.0001), PS (*p* = 0.0045, *p* = 0.0001) and AmB group (*p* = 0.0073, *p* = 0.0001) (Figure 2B). Moreover, mitochondrial activity of PS-AmB-Cu^2+^-treated cells was significantly higher than that measured for AmB and PS-treated cells (*p* = 0.0308 and *p* = 0.0047, respectively). 

After 72 h, the cells that were exposed to AmB and PS-AmB showed a significant increase in cell viability compared to the control, PS-, AmB-Cu^2+^-, and PS-AmB-Cu^2+^-treated cells (*p* < 0.0080 in all comparisons) (Figure 2C). Viability was also significantly increased in cells treated with PS-AmB-Cu^2+^ comparing to the control and cells exposed to PS (*p* < 0.0030 in both comparisons) (Figure 2C). 

### 2.3. Impact of Antibiotics on the Mesenchymal Stem Cells Markers

Besides direct effects on cell viability, antibiotics may also affect the stemness phenotype of ADSC. Hence, we have assessed how the tested combinations of antibiotics may affect the expression of mesenchymal stem cell markers like CD73, CD90, and CD105, both at mRNA and protein level. 

None of the tested drugs or their combinations has changed the expression of *CD90* mRNA in comparison to control. AmB caused significantly lower mRNA expression of CD90 marker compared to PS (*p* = 0.0086). 

**Figure 3:**
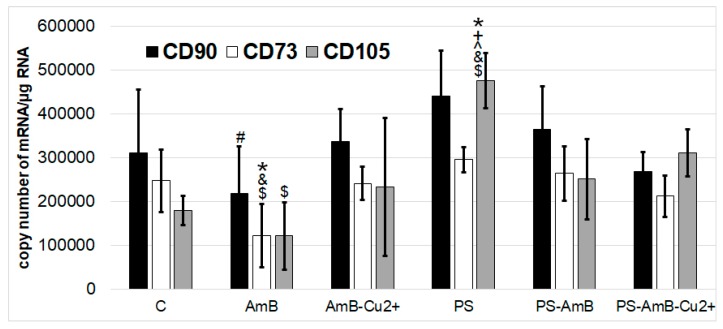
The mRNA levels of MSC markers *CD73*, *CD90* and *CD105* in the ADSC after exposure to the tested antibiotic combinations (RT-qPCR analysis). The bars represent the means ± standard deviation (SD) of the copy numbers per 1 µg of total RNA; ANOVA with the Tukey post hoc test, * *p* < 0.05 vs. control, ^#^ *p* < 0.05 vs. PS, ^ *p* < 0.05 vs. AmB-Cu^2+^, ^+^ *p* < 0.05 vs. AmB, ^&^ *p* < 0.05 vs. PS-AmB, ^$^ *p* < 0.05 vs. PS-AmB-Cu^2+^.

AmB caused also decrease of *CD73* mRNA level comparing to control, PS-, AmB-Cu^2+^-, and PS-AmB-treated cells (*p* = 0.0063, *p* = 0.0002, *p* = 0.0098, *p* = 0.0016, respectively) (Figure 3). 

*CD105* mRNA expression was statistically significantly higher after treatment with PS comparing to control, AmB-, AmB-Cu^2+^-, PS-AmB- and PS-AmB-Cu^2+^-treated cells (*p* = 0.0002, *p* = 0.0001, *p* = 0.0008, *p* = 0.0136, *p* = 0.0331, respectively). AmB caused decrease of *CD105* mRNA level comparing PS-AmB-Cu^2+^ (*p* = 0.0100). 

**Figure 4:**
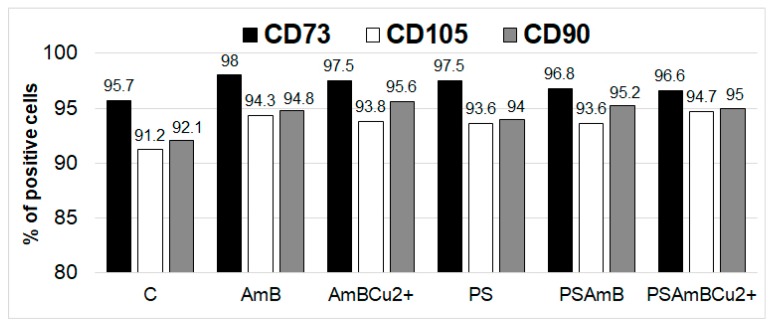
The percentage of positive cells for CD73, CD90 and CD105 markers after treatment with tested antibiotic and their combinations (FACS analysis).

Fluorescence-activated cell sorting (FACS) analysis showed the presence of CD73, CD90 and CD105 markers at protein level in all analyzed groups. The percentage of marker-positive cells was slightly higher after treatment with antibiotics (Figure 4).

**Figure 5:**
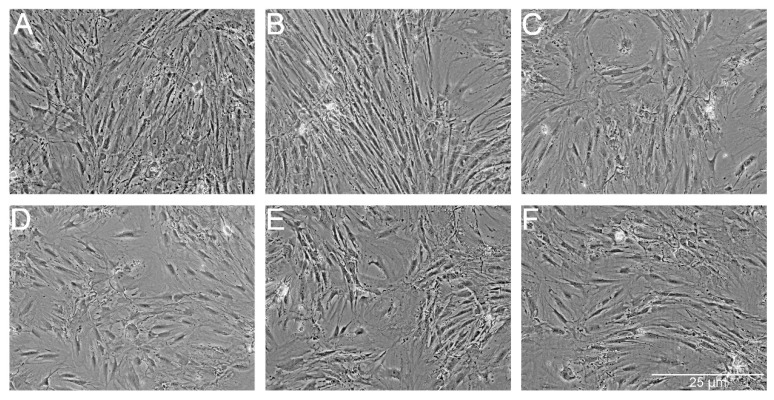
The morphology of the ADSC after exposure to the tested combinations of antibiotics. ADSC were exposed for 14 days to AmB (**B**), AmB-Cu^2+^ (**C**), PS (**D**), PS-AmB (**E**) and PS-AmB-Cu^2+^ (**F**), and compared to the control cells (**A**).

We also analyzed the morphology of the adipose-derived stem cells after 14 days (Figure 5). We did not observe any essential differences because all of the cells retained their fibroblast-like shape. 

### 2.4. Impact of Antibiotics on Adipogenesis

**Figure 6:**
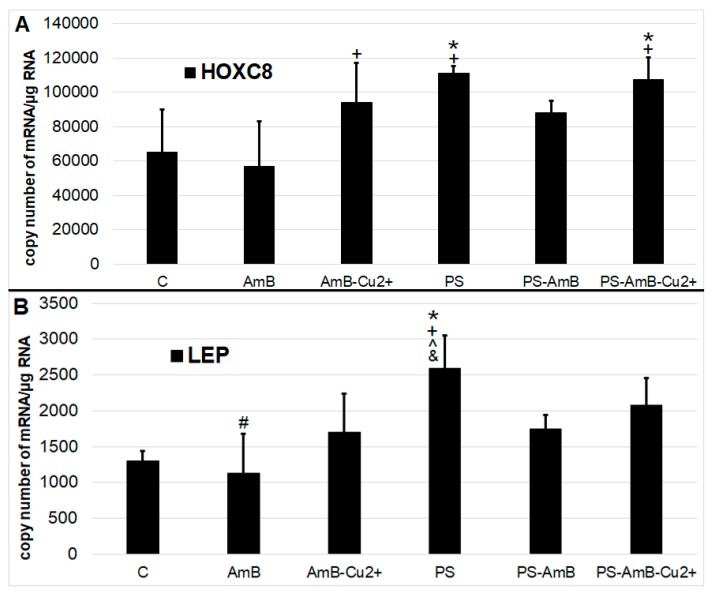
The mRNA levels of adipose tissue markers ((**A**)—*HOXC5*; (**B**)—*LEP*). The bars represent the means ± standard deviation (SD) of the copy numbers per 1 µg of total RNA; ANOVA with the Tukey post hoc test, * *p* < 0.05 vs. control, ^#^ *p* < 0.05 vs. PS, ^ *p* < 0.05 vs. AmB-Cu^2+^, ^+^ *p* < 0.05 vs. AmB, ^&^ *p* < 0.05 vs. PS-AmB.

Antibiotics may also induce differentiation towards certain lineage commitments. Hence, to examine that possibility, cells were cultured for 14 days in standard medium without dedicated factors stimulating adipogenesis. We have analyzed adipogenesis markers—*HOXC8* (Figure 6A) and *LEP* genes (Figure 6B)—at the mRNA level using RT-qPCR. 

The expression of *HOXC8* gene was increased after exposure to PS and PS-AmB-Cu^2+^, compared to the control (*p* = 0.0071 and *p* = 0.0067, respectively). In AmB-treated cells, the expression of this marker was significantly lower comparing to PS, AmB-Cu^2+^ and PS-AmB-Cu^2+^ (*p* = 0.0012, *p* = 0.0426 and *p* = 0.0010, respectively). 

The level of *LEP* mRNA was significantly higher in cells exposed to PS in comparison to control, AmB-, AmB-Cu^2+^-, and PS-AmB-treated cells (*p* = 0.0005, *p* = 0.0001, *p* = 0.0094, and *p* = 0.0150, accordingly). Additionally, in cells treated with AmB, the *LEP* mRNA level was significantly lower than in PS-AmB-Cu^2+^-treated cells (*p* = 0.0053).

**Figure 7:**
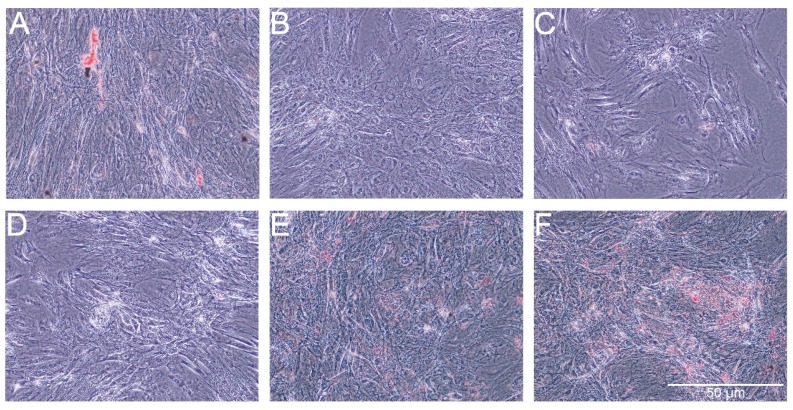
Oil red staining of lipid droplets after exposure of adipose-derived stem cells (ADSC) to the tested combinations of antibiotics. ADSC were exposed for 14 days to AmB (**B**), AmB-Cu^2+^ (**C**), PS (**D**), PS-AmB (**E**) and PS-AmB-Cu^2+^ (**F**), and compared to the control cells (**A**).

The Oil Red O showed very weak staining of lipids in control cells and slightly stronger staining in cells treated with PS-AmB-Cu^2+^ (Figure 7). The optical density (OD) value was the highest for PS-AmB-Cu^2+^-treated cells (OD = 0.65). 

### 2.5. Impact of Antibiotics on Nonstimulated Osteogenesis

**Figure 8:**
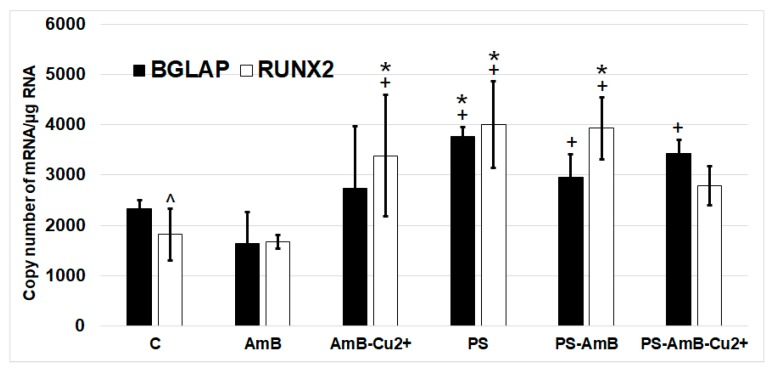
The mRNA levels of osteoblastic markers *BGLAP* and *RUNX2* in the ADSC after exposure to the tested antibiotic combinations. The bars represent the means ± standard deviation (SD) of the copy numbers per 1 µg of total RNA; ANOVA with the Tukey post hoc test, * *p* < 0.05 vs. control, ^+^ *p* < 0.05 vs. AmB, ^ *p* < 0.05 vs. AmB-Cu^2+^.

To investigate possible effects of the tested combinations of antibiotics towards osteoblastic differentiation, we have assessed the changes in the expression of osteoblastic markers (*BGLAP*, *RUNX2*, *ALP* and *SPP1* mRNAs) by RT-qPCR. The ADSC were cultured for 14 days in standard Dulbecco’s Modified Eagle Medium (DMEM) without dedicated factors stimulating osteoblastic differentiation. The changes in the expression of osteoblast markers *BGLAP* and *RUNX2* were similar in studied groups (Figure 8). *ALP* and *SPP1* mRNA was not detectable in analyzed groups.

The expression of *RUNX2* was significantly higher in cells treated with PS, AmB-Cu^2+^ and PS-AmB in comparison to control (accordingly: *p* = 0.00003, *p* = 0.0110, *p* = 0.0005). AmB caused downregulation of this marker in comparison to PS-, AmB-Cu^2+^- and PS-AmB-treated cells (*p* = 0.0014, *p* = 0.0279, *p* = 0.0020, respectively). 

*BGLAP* mRNA level was higher in PS-treated cells comparing to control (*p* = 0.0079). After AmB treatment, the expression of *BGLAP* was lower in comparison to groups: PS, PS-AmB and PS-AmB-Cu^2+^ (*p* = 0.0002, *p* = 0.0181, *p* = 0.0008, respectively). 

**Figure 9:**
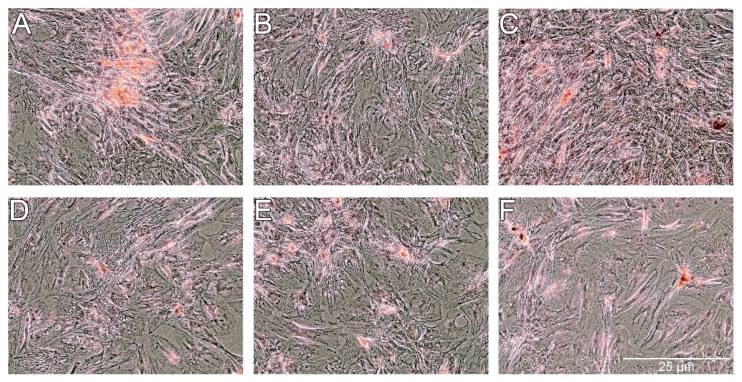
Alizarin Red staining of extracellular calcium after exposure of ADSC to the tested combinations of antibiotics. ADSC were exposed for 14 days to AmB (**B**), AmB-Cu^2+^ (**C**), PS (**D**), PS-AmB (**E**) and PS-AmB-Cu^2+^ (**F**), and compared to the control cells (**A**).

Alizarin Red staining showed slight increase of extracellular calcium in the groups of cells treated with AmB-Cu^2+^, PS and PS-AmB (Figure 9). However, significant difference was statistically confirmed only in the comparison between AmB-Cu^2+^ and PS-AmB-Cu^2+^ groups (*p* = 0.0380).

### 2.6. Impact of Antibiotics on Osteogenesis in the Presence of Pro-Osteogenic Factors

**Figure 10:**
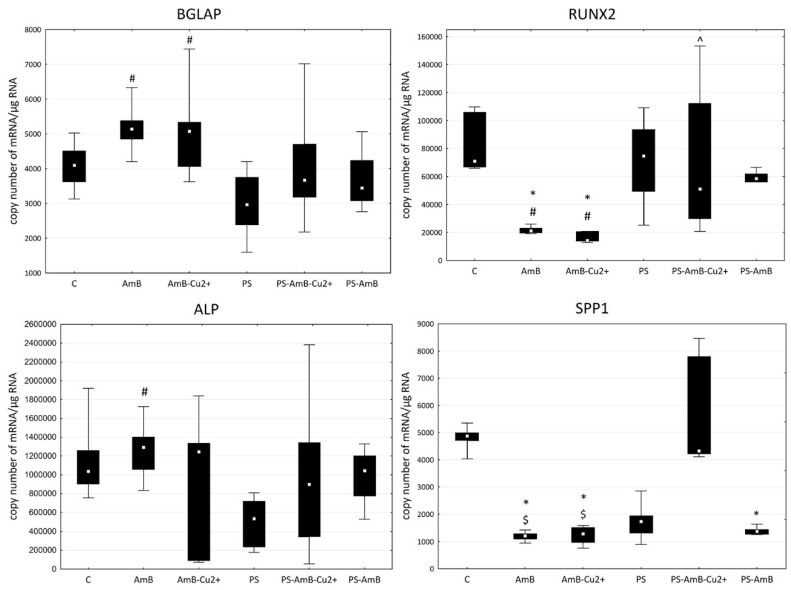
The mRNA levels of osteogenic markers *BGLAP*, *RUNX2*, *SPP1* and *ALP* in the ADSC after exposure of ADSC to the tested combinations of antibiotics. The bars represent the (Me) with the 25th and 75th quartiles and the minimum and maximum of the copy numbers per 1 µg of total RNA; the Kruskal–Wallis test with post hoc was applied to assess any differences in the expression of the genes, * *p* < 0.05 vs. control, ^ *p* < 0.05 vs. AmB-Cu^2+^, ^#^ *p* < 0.05 vs. PS, ^$^ *p* < 0.05 vs. PS-AmB-Cu^2+^.

Following the results shown above, we decided to perform follow-up experiments to test if/how the tested antibiotics affect stimulated osteogenic differentiation. To induce osteoblastic differentiation, the ADSC were cultured for 14 days in a DMEM medium supplemented with factors that stimulate osteogenesis (L-ascorbic acid 2-phosphate, dexamethasone and β-glycerophosphate) and combinations of tested antibiotics (Figure 10). 

Expression of *BGLAP* mRNA did not differ in comparison to the control. However, there was statistically significantly higher expression in AmB- (*p* = 0.0023) and in AmB-Cu^2+^- (*p* = 0.0091) compared to PS-treated cells.

The mRNA level of *RUNX2* was significantly lower in cells treated with AmB and AmB-Cu^2+^ compared to the control (*p* = 0.0019 and *p* = 0.0002) and PS-treated cells (*p* = 0.0134 and *p* = 0.0016). Moreover, the *RUNX2* expression in AmB-Cu^2+^ was significantly lower than in cells treated with PS-AmB-Cu^2+^ (*p* = 0.0154).

*SPP1* mRNA showed a statistically significantly lower expression in groups: AmB (*p* = 0.0002), AmB-Cu^2+^ (*p* = 0.0033) and PS-AmB (*p* = 0.0334), compared to control. There was also a statistically significantly lower expression of this marker in AmB- (*p* = 0.0005226) and AmB-Cu^2+^ groups (*p* = 0.008530) in comparison to PS-AmB-Cu^2+^-treated cells. 

No statistically significant difference was observed in the expression of *ALP* in the examined groups in comparison to control cells. The expression of *ALP* was significantly higher in AmB-treated cells compared to group PS (*p* = 0.0123). 

**Figure 11:**
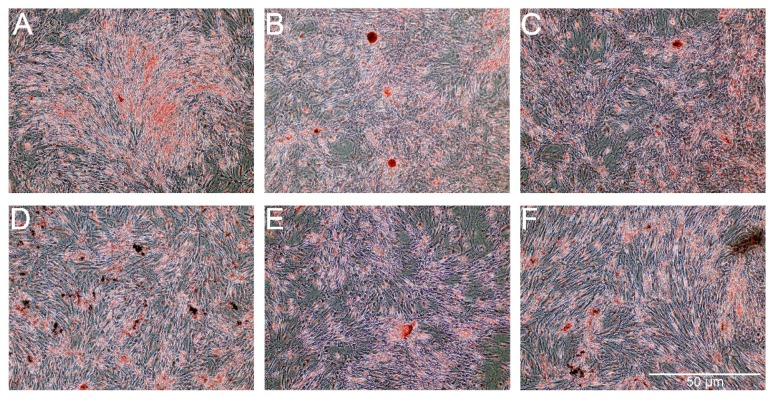
The Alizarin Red staining in the ADSC after exposure to the osteogenesis medium with the addition of AmB (**B**), AmB-Cu^2+^ (**C**), PS (**D**), PS-AmB (**E**) and PS-AmB-Cu^2+^ (**F**) compared to the control cells (**A**).

For osteogenesis, the presence of extracellular calcium was confirmed by Alizarin Red staining. As shown in Figure 11, the presence of extracellular calcium was observed in all of the groups of cells. A lower level of calcium content was noticed for cells treated with PS and PS-AmB-Cu^2+^ compared to control (*p* = 0.0001 and *p* = 0.0002). Calcium level was significantly higher for cells treated with AmB in comparison to control cells (*p* = 0.0015) and cells treated with PS (*p* = 0.0001), PS-AmB (*p* = 0.0030), and PS-AmB-Cu^2+^ (*p* = 0.0001). AmB-Cu^2+^ and PS-AmB caused increase of calcium content in comparison to PS- (*p* = 0.0001 and *p* = 0.0001) and PS-AmB-Cu^2+^-treated cells (*p* = 0.0001 and *p* = 0.0007).

## 3. Discussion

Mesenchymal stem cells (MSC) are multipotent, somatic cells with multilineage differentiation ability. Furthermore, they also have immunomodulatory, angiogenic and antiapoptotic activities through the secretion of growth factors, cytokines and peptides. MSC can be isolated from bone marrow, skin, whole blood or adipose tissue. ADSC have many advantages such as a low risk of side effects when being harvested and the fact that the tissue is easy to obtain from almost every patient, or from medical waste upon cosmetic surgeries; hence there are no ethical issues. Moreover, the cells are easy to culture and they can differentiate into adipocytes, chondrocytes, osteoblasts and myocytes [2,5,6,8,20]. 

Many endogenous and exogenous factors may affect the proliferation and differentiation ability of mesenchymal stem cells, e.g., derivation of adipose tissue, age of donors, comorbidities, method used to isolate the stem cells, as well as the duration and conditions of culturing them [20,21]. The role of the antibiotics is to prevent any contamination of the cell culture. However, it is necessary to only use them temporarily because longer exposures may lead to important phenotype changes. Additionally, some antibiotics and especially antimycotics can also be cytotoxic. Cohen et al. proved, for example, that gentamycin and penicillin-streptomycin inhibit the growth of embryonic stem cells [13]. 

Moreover, all methods of nonisogenic transplantation require the use of immunosuppressive agents, which induces a temporary immune deficiency, often facilitating dangerous fungal infections. The types of antifungal drugs that are used depend on an individual’s symptoms, concurrent illnesses and the species of fungi [10,22]. The main groups of antifungal drugs that are used in patients with hematopoietic stem cell transplantations are azoles such as fluconazole, echinocandins and polyenes, e.g., different forms of amphotericin B (AmB), a drug that is also most often used to prevent a fungal contamination in a cell culture. Antifungal activities are arising from the inhibition of the synthesis of nucleic acids due to their effect on the ergosterol in the liquid membrane [10,22]. Unfortunately, AmB’s clinical use is limited by its strong nephrotoxic properties [23]. The mechanism of the nephrotoxicity of AmB is not well-known. It is possibly connected to the formation of pores and the destabilization of the cell membrane [15,23,24]. Furthermore, AmB induces the expression of cytokines and chemokines that are involved in destroying the kidneys, e.g., a higher expression of interleukin 1β, TNFα, IL-6 and IL-8 [24]. The aim of many studies has been the creation of a new form of amphotericin B, e.g., such as a lipid complex or liposomal and colloid forms [10,24,25,26], to counteract its limitations. A recently developed complex of amphotericin B with copper (II) ions possess high antifungal properties and is less toxic to animal cells than AmB [16,17,18]. However, its activity on MSC has not been studied so far.

To gain better understanding of the used experimental systems in regenerative medicine and tissue engineering, we have evaluated the effect of antibiotics on the growth and differentiation of the MSC derived from the adipose tissue commonly used in cell culture settings and in the clinic. Additionally, previous studies reporting lower toxicity of AmB-Cu^2+^ on human proximal tubular epithelial cells (RPTEC) and fibroblasts encouraged us to assess also its influence on mesenchymal stem cells [17,18].

Our experiments examined the short- and long-term effects of antibiotics and antimycotics. The concentrations of penicillin-streptomycin were determined based on literature data [10,11]. In a previous study, it was proven that 0.5 μg/mL of AmB and AmB-Cu^2+^ did not inhibit the growth of RPTECs [18]. This concentration was selected as suitable for the analysis. It is also the most often used concentration of AmB in cell cultures of mesenchymal stem cells, especially for adipose-derived stem cells, according to the literature. Initially, we analyzed the influence of antibiotics and antimycotics on the growth of adipose-derived stem cells. The addition of antibiotics adversely affected cell growth within 24 h. Similar results were obtained by others while testing the effects of rifampicin and chloramphenicol [27]. Both antibiotics reduced the cell growth in limbal stem cells [27]. We however did not observe any substantial inhibition of the mitochondrial activity using an MTT assay; surprisingly, there was higher mitochondrial activity in the cells after exposure to AmB-Cu^2+^. After 48 h, we observed similar results in both assays (cell growth SRB—sulforhodamine B and MTT), and the highest proliferation of the cells occurred in the group that was treated with AmB-Cu^2+^. After 72 h however, an inhibition of protein production and mitochondrial activity was detected in the cells after exposure to AmB-Cu^2+^ compared to AmB alone. As the first days of the experiment caused an increased proliferation, the decrease observed after 72 h may be the result of the rapid depletion of nutrients in the culture medium of cell treated with AmB-Cu^2+^. In turn, observed increase of cells viability after 72 h of incubation with AmB may indicate that MSC inactivated this drug only after two days. Thus, we conclude that MSC tolerate the exposure to AmB-Cu^2+^ much better than AmB. Our finding in these assays indicates that the cellular changes depend on the time of exposure and type, as well as combination of antibiotics used in the experiment. Some discrepancies observed between the read-outs from SRB and from the MTT assay reflect the fact that both assays measure different cellular parameters.

We have also made some interesting observations regarding the effects of tested combinations of antibiotics on the maintenance of MSC phenotype, as assessed by the expression of various stemnesses and differentiation markers. ADSC were cultivated in standard medium without factors stimulating differentiation into adipocytes or osteocytes. We noticed a slight increase of population of cells coexpressing MSC markers under exposure to tested drugs. However, AmB caused a decrease of MSC markers at mRNA level. This may result from the influence of amphotericin B on mRNA stability. 

To assess potential adipogenic differentiation, we measured the expression (RT-qPCR) of *HOXC8* (*homebox 8*) and *LEP* (*leptin*) genes. These genes are characteristic for white adipose tissue cells [28]. To some degree, all the combinations of tested antibiotics, except AmB, did increase the expressions of *LEP* and *HOCX8*, most significantly the penicillin-streptomycin. However, lipid staining with Oil Red O resulted in a weak signal, except for cells treated with PS-AmB-Cu^2+^. Hence, the addition of PS may be beneficial in supporting adipogenic differentiation in desirable medical settings. On the other hand, some studies have proved that PS may be cytotoxic to embryonic stem cells. Furthermore, Zur Nieden and colleagues have reported that penicillin G inhibited the differentiation of embryonic stem cells into osteocytes and chondrocytes [29]. Thus, we assessed also the influence of tested drugs on osteogenesis. 

All antibiotics, except AmB, caused an increase of *RUNX2* and *BGLAP* mRNA level. We did not detect the mRNA of *ALP* and *SPP1*. However, the staining with Alizarin Red revealed the presence of extracellular calcium content, therefore we decided to evaluate the influence of tested drugs on osteogenesis in the presence of dedicated factors stimulating this process. We observed significant differences between amphotericin B and penicillin-streptomycin on the induction of osteogenesis. This study showed that amphotericin B and its new form with copper ions did produce significant effects of the osteogenesis, especially the lower expression of *RUNX2*, which is an early marker of osteogenesis and the *osteopontin* (*SPP1*) gene, which is a late marker of osteogenesis. Interestingly, we did observed a higher expression of the gene coding osteocalcin (*BGLAP*), which is a medium marker of osteogenesis. Hence, we can assume that amphotericin B and AmB-Cu^2+^ influence osteogenesis in the presence of osteogenesis-induction factors, including dexamethasone, β-glycerophosphate and L-ascorbic acid. The combination of AmB or AmB-Cu^2+^ with penicillin-streptomycin markedly modulated the expression of osteogenesis markers. Alizarin Red staining confirmed that AmB promoted osteogenesis, while addition of penicillin-streptomycin caused weaker signal. The mechanism of the action of streptomycin is connected to its binding to the 30S subunit of the bacterial ribosome. However, it also possesses some affinity to the ribosomes in eukaryotic cells. Relier and colleagues suggested that streptomycin may act on eukaryotic cells through the disruption of protein synthesis, by affecting mitochondrial activity, by binding to certain RNAs or by interfering with miRNAs action [12]. This indicates that the addition of streptomycin changes the gene expression profile in cells and it may affect the differentiation process. 

## 4. Material and Methods

### 4.1. Cell Culture 

Normal human adipose-derived stem cells (ADSC) (PT-5006; Lonza, Basel, Switzerland) were routinely maintained in a DMEM medium (Dulbecco’s Modified Eagle Medium, Lonza, Basel, Switzerland), which was supplemented with fetal bovine serum (FBS, EuroClone, Pero, Italy), at 37 °C in a 5% CO_2_ incubator (Direct Heat CO_2_; Thermo Fisher Scientific, Waltham, MA, USA). The ADSC that were used for the experiment were at second passage.

### 4.2. Exposure of Cells to Antibiotics

The cells were treated with 0.5 µg/mL of amphotericin B (AmB) (Sigma-Aldrich, St. Louis, MO, USA), 0.5 µg/mL of amphotericin B complex with copper ions (AmB-Cu^2+^) [16,30], 10 µL/mL of penicillin-streptomycin complex (PS) (100 IU/mL for penicillin and 100 μg/mL for streptomycin) (Lonza) or combinations of these—amphotericin B with penicillin-streptomycin (PS-AmB) and the AmB-Cu^2+^ with penicillin streptomycin (PS-AmB-Cu^2+^). Control cells were cultured in a medium without antibiotics. 

### 4.3. Sulforhodamine B Assay 

The cells were seeded into a 96-well culture plate at a density of 5 × 10^3^ cells per well. The cellular protein content in the cells after exposure to antibiotics was tested using a Sulforhodamine B (SRB)-based in vitro toxicology assay kit (TOX6, Sigma-Aldrich, St Louis, MO, USA) and a Wallac 1420 VICTOR plate reader (Perkin Elmer, Waltham, MA, USA) according to the manufacturer’s instructions. The total cellular protein content in the cells was measured after 24 h, 48 h and 72 h of exposure.

### 4.4. MTT Assay

For the cytotoxicity assay, the cells were seeded into a 96-well culture plate at a density of 5 × 10^3^ cells per well. MTT-assay was performed similarly as described recently [31]. Briefly, 3-[4,5-dimethylthiazol-2-yl]-2,5-diphenyltetrazolium bromide (MTT) (Sigma-Aldrich, St. Louis, MO, USA) conversion was used to evaluate the metabolic activity of the cells after their exposure to antibiotics, according to the manufacturer’s instructions. Absorbance was measured using Wallac 1420 VICTOR plate reader (Perkin Elmer, Waltham, MA, USA). The mitochondrial activity of the cells was measured after 24 h, 48 h and 72 h of exposure. 

### 4.5. Quantitative Real-Time Polymerase Chain Reaction Assay 

Total RNA was extracted from the cells using a TRIzol reagent (Invitrogen, Carlsbad, CA, USA) according to the manufacturer’s instructions. The nucleic acid concentration was determined using a MaestroNano Spectrophotometer (Maestrogen, Hsinchu City, Taiwan). The expression mRNAs was detected using the real time RT-qPCR technique with SYBR Green chemistry (SYBR Green Quantitect RT-PCR Kit, Qiagen, Hilden, Germany) and an Opticon™ DNA Engine Continuous Fluorescence detector (MJ Research Inc., Waltham, MA, USA) as was previously described [32]. All of the samples were tested in triplicate. β-actin was included as an endogenous positive control (housekeeping/structural gene) of the amplification and the integrity of the extracts. Oligonucleotide primers were purchased from the Sigma Aldrich Company (Sigma-Aldrich, St. Louis, MO, USA). Each run was completed using a melting curve analysis to confirm the specificity of the amplification and the absence of primer dimers. The RT-qPCR products were separated on 6% polyacrylamide gels and visualized using silver salts. To quantify the results for the products that were obtained by RT-qPCR, the standard curve method was used as was previously described [32]. 

### 4.6. Mesenchymal Stem Cells Markers Analysis 

Cells were cultured for 14 days in medium without any differentiation factors in the presence of tested antibiotics and their combinations. Control cells were cultured in the medium without antibiotics. Cells were collected to the RT-qPCR analysis and for flow cytometry analysis (CD73, CD90, CD105). R&D Systems Human Mesenchymal Stem Cell Marker Verification Multi-Color Flow Cytometry Kit (R&D Systems, Minneapolis, MN, USA) was used for mesenchymal stem cells markers identification: CD73-CFS, CD90-APC, CD105-PerCP. Cell fluorescence was measured immediately after staining (FACS Aria 2; Becton Dickinson, Franklin Lakes, NJ, USA), and data were analyzed using software (Becton Dickinson, Franklin Lakes, NJ, USA) measured as the percentile of gated cells in fluorescent channels with activities of the corresponding isotype controls.

### 4.7. Adipocyte Differentiation Analysis without Differentiation Factors

After 14 days of culture, the Oil Red O staining and RT-qPCR analysis of HOX2 and LEP mRNAs was performed.

Oil Red O staining is a microscopic visualization of the lipid droplets that are characteristic structures for adipocytes. For quantification of staining after washing out unbound dye and extraction with the isopropanol from cells, spectrophotometric analysis for quantitative assessment was carried out. The obtained absorbance value was calculated based on a calibration curve prepared with a series of Oil Red O solutions of known concentrations. The OD of supernatants was read at 405 nm in 96-well plate using a Perkin Elmer Wallac VICTOR2.

### 4.8. Osteoblast Differentiation Analysis without Differentiation Factors

After 14 days of cell culture without any differentiation factors, Alizarin Red staining and RT-qPCR analysis (*BGLAP*, *RUNX2*, *ALP*, *SPP1* mRNAs) was performed.

Alizarin Red staining was used to evaluate calcium deposits in the culture by the cells. Alizarin Red at a concentration 40 mM was prepared in ddH_2_O and the pH was adjusted to 4.2 using 10% ammonium hydroxide. The assayed cells were washed with phosphate buffered saline (PBS) and fixed in 70% ethanol (Sigma-Aldrich, St Louis, MO, USA). Next, the monolayers were washed twice with dH_2_O and then Alizarin Red S was added. The plates were incubated at room temperature for 30 min with shaking. Stained cells were visualized using an Olympus IX81 microscope (Shinjuku, Tokyo, Japan) and a DP70 camera (Olympus, Shinjuku, Tokyo, Japan) was used for the photographic documentation. To quantify the staining, the acetic acid (Sigma-Aldrich, St Louis, MO, USA) method was applied to recover the stain from calcium deposits. Stain solutions were adjusted to pH 4.2 with ammonium hydroxide and samples were read at 405 nm in a 96-well plate using a Perkin Elmer Wallac VICTOR2.

### 4.9. Osteoblast Differentiation Analysis in the Presence of Differentiation Factors

Next, the cells were plated and grown until there was 80% of the confluent after which the medium was replaced with a fresh DMEM medium with fetal bovine serum (FBS) and 0.2 mM L-ascorbic acid 2-phosphate, 10^7^ M dexamethasone and 10 mM β-glycerophosphate (Sigma-Aldrich, St Louis, MO, USA). Cells were cultured in the above-described media with or without antibiotics for another 14 days. After 14 days, the cells were collected and the Alizarin S red staining and RT-qPCR analysis (*BGLAP*, *RUNX2*, *ALP*, *SPP1* mRNAs) was performed. 

### 4.10. Statistical Analysis

Statistical analysis was performed using Statistica 13.0 software (StatSoft, Tulsa, OK, USA). The values are expressed as the means and standard deviation (SD) for normally distributed data. A one-way ANOVA test and a Tukey’s post hoc test were applied to evaluate any differences in the examined groups. For the non-normally distributed data, values were expressed as median value (Me) with the 25th and 75th quartiles and the Mann–Whitney U test was applied to assess differences in the expression of genes. Differences between the different groups were compared using Kruskal–Wallis test. The level of significance was set at *p* < 0.05 for all of the statistical tests. 

## 5. Conclusions

In summary, our results suggest that the presence of the antibiotics that are commonly used in cell cultures is not completely neutral to the phenotype of ADSC. Moreover, they modulate the differentiation process. Both AmB and AmB-Cu^2+^ as well as their combinations with PS affect the level of differentiation markers, however AmB-Cu^2+^ is better tolerated by stem cells. Antibiotics should probably only be used in special situations such as the isolation of a cell from a biopsy and at the early stages of a culture after which a standard culture should be done without this addition. Finally, it would be advised to choose the combination of antibiotics and antimycotics with caution, in a more targeted fashion, when propagating ADSC and possibly also other cell types. The findings are especially important given a rapid increase of experimental use of stem cells, in particular induced-pluripotent stem cells (iPS), and MSC (often ADSC), in the development of various regenerative medicine techniques [33,34,35]. Rapid development of direct transdifferentiation techniques, which bypass the stage/use of stem cells [1,36,37,38], is another area where the presented in this manuscript results may be of key importance for the optimal results.

## Abbreviations

ADSC	adipose-derived stem cells
ALP	alkaline phosphatase
AmB	amphotericin B
AmB	Cu^2+^-complex of AmB with copper(II) ions
ANOVA	analysis of variance
ATCC	American Type Culture Collection
BGLAP	osteocalcin
bis-AAF-R110	bis-alanyl-alanyl-phenylalanyl-rhodamine 110
CD105	Endoglin
CD73	cluster of differentiation 73/ecto-5′-nucleotidase
CD90	cluster differentiation 90/Thy-1 antigen
dH_2_O	distilled water
DMEM	Dulbecco’s Modified Eagle Medium
FBS	fetal bovine serum
GF-AFC	glycyl-phenylalanyl-aminofluorocoumarin
HOXC8	homebox 8
IL-6	interleukin 6
IL-8	interleukin 8
LEP	leptin
mRNA	messenger ribonucleic acid
MSC	mesenchymal stem cells
MTT	3-[4,5-dimethylthiazol-2-yl]-2,5-diphenyltetrazolium bromide
PBS	phosphate buffered saline
PS	penicillin-streptomycin complex
PS-AmB	amphotericin B with penicillin-streptomycin
PS-AmB-Cu^2+^	amphotericin B complex with copper (II) ions with penicillin streptomycin
RFU	relative fluorescence units
RLU	relative luminescence units
ROS	reactive oxygen species
RTq PCR	quantitative real-time polymerase chain reaction
RUNX2	runt-related transcription factor 2
SPP1	osteopontin
SRB	sulforhodamine B
SVF	stromal vascular fraction
TNFα	tumor necrosis factor α.
